# Thyroid Function after Subtotal Thyroidectomy in Patients with Graves' Hyperthyroidism

**DOI:** 10.1100/2012/548796

**Published:** 2012-02-01

**Authors:** E. J. Limonard, P. H. Bisschop, E. Fliers, E. J. Nieveen van Dijkum

**Affiliations:** ^1^Department of Surgery, Academic Medical Center, University of Amsterdam, Meibergdreef 9, 1105 AZ, Amsterdam, The Netherlands; ^2^Department of Endocrinology and Metabolism, Academic Medical Center, University of Amsterdam, Meibergdreef 9, 1105 AZ, Amsterdam, The Netherlands

## Abstract

*Background*. Subtotal thyroidectomy is a surgical procedure, in which the surgeon leaves a small thyroid remnant in situ to preserve thyroid function, thereby preventing lifelong thyroid hormone supplementation therapy. *Aim*. To evaluate thyroid function after subtotal thyroidectomy for Graves' hyperthyroidism. *Subjects and Methods*. We retrospectively reviewed the medical records of all patients (*n* = 62) who underwent subtotal thyroidectomy for recurrent Graves' hyperthyroidism between 1992 and 2008 in our hospital. Thyroid function was defined according to plasma TSH and free T4 values. *Results*. Median followup after operation was 54.6 months (range 2.1–204.2 months). Only 6% of patients were euthyroid after surgery. The majority of patients (84%) became hypothyroid, whereas 10% of patients had persistent or recurrent hyperthyroidism. Permanent recurrent laryngeal nerve palsy and permanent hypocalcaemia were noted in 1.6% and 3.2% of patients, respectively. *Conclusion*. In our series, subtotal thyroidectomy for Graves' hyperthyroidism was associated with a high risk of postoperative hypothyroidism and a smaller, but significant, risk of persistent hyperthyroidism. Our data suggest that subtotal thyroidectomy seems to provide very little advantage over total thyroidectomy in terms of postoperative thyroid function.

## 1. Introduction

Graves' disease is the most common cause of hyperthyroidism [1]. Three treatment modalities are available for the treatment of hyperthyroidism: antithyroid medication, radioiodine therapy, and surgery. According to the recent guidelines on the treatment of hyperthyroidism from The American Thyroid Association and The American Association of Clinical Endocrinologists [2], patients with Graves' hyperthyroidism can be treated with any of these treatment options. Factors favoring a particular treatment and contraindications for certain treatment options are discussed in the guidelines. Physicians should inform patients on potential side effects and benefits of each option before making a decision. There is a wide geographic variation in the choice of therapy [3–6]. Medical treatment with antithyroid drugs is often accepted as first-choice modality in Europe, followed by radioiodine in case of recurrence. Although surgery offers the advantage of quick control and low morbidity in experienced hands, it is infrequently recommended as initial treatment. Surgery is indicated for recurrent Graves' disease, when other therapeutic options are contraindicated and in patients with large goiters.

Two different surgical techniques are used for the treatment of Graves' hyperthyroidism: a total thyroidectomy (TT) and a subtotal thyroidectomy (STT), which leaves a small unilateral or bilateral remnant in situ. While there is general consensus that total thyroidectomy is the procedure of choice in patients with thyroid carcinoma, the optimal surgical approach for benign thyroid disease has remained controversial. The main reason for performing a subtotal thyroidectomy is a presumably lower incidence of postoperative complications, including recurrent laryngeal nerve (RLN) paralysis and hypoparathyroidism, and an anticipated postoperative euthyroid state by leaving a small remnant of thyroid tissue in situ to maintain adequate hormone production. There is, however, a risk that the disease will persist or recur in the remnant. More recent studies favoured TT as preferred procedure in the surgical management of Graves' disease, because the residual function after STT was not as good as previously believed and the complication rates were not different between the TT and STT procedure [7–10].

The aim of this retrospective study was to evaluate the long-term treatment outcome of patients treated with subtotal thyroidectomy for Graves' disease in our institution. We recorded postoperative thyroid function, along with the postoperative complication rates, to determine if subtotal thyroidectomy should still play a role in the surgical management of Graves' hyperthyroidism.

## 2. Methods

### 2.1. Patients

All operation records of patients surgically treated for thyroid disease between January 1992 and December 2008 were reviewed to identify patients treated with subtotal thyroidectomy for Graves' disease. Patients were diagnosed with Graves' disease based on patient history, clinical examination, hormonal and/or immunologic assays, and thyroid scintigraphy. Charts, medical records, and correspondence of all departments involved in the treatment were retrospectively reviewed for data collection.

### 2.2. Procedure and Followup

Patients were pretreated with antithyroid medication and/or with amiodarone, ipodate, or Lugol's solution to achieve euthyroidism on the day of surgery.

Most operations before 2000 were performed by the same surgeon; thereafter, two additional surgeons also operated these patients. All three surgeons had extensive experience in thyroid surgery. For each subtotal thyroidectomy, a standard surgical technique was used. All patients underwent a unilateral subtotal hemithyroidectomy, leaving approximately 5–8 grams of thyroid tissue, and a contralateral total hemithyroidectomy.

Pre- and postoperatively, patients underwent direct laryngoscopy to check for vocal cord paralysis. Recurrent laryngeal nerve palsy was defined as permanent if it persisted more than 12 months postoperatively. Serum calcium levels were routinely measured four hours after surgery and at least once daily during hospital stay. Hypocalcaemia was defined as a serum calcium level <2.20 mmol/L (reference range 2.20–2.60 mmol/L). Oral or intravenous calcium and/or calcitriol suppletion was given when serum calcium level was <1.80 mmol/L and/or when symptoms developed. Permanent hypoparathyroidism was defined as requirement of calcitriol supplementation to maintain normal serum calcium levels 12 months after surgery.

Postoperatively, patients were assessed clinically and biochemically. Thyroid status was determined by measuring plasma thyroid-stimulating hormone (TSH): reference values 0.40–4.00 mU/L until 2007 and 0.50–5.00 mU/L as of 2008, free thyroxine (FT4): reference values 10–20 pmol/L until 1995 and 10–23 pmol/L as of 1996, and triiodothyronine (T3): reference values 1.30–2.70 nmol/L. Patients were classified as postoperative hypothyroid, in the case of FT4 levels below the reference range and/or elevated TSH levels on at least two consecutive measurements. These patients were prescribed thyroid hormone replacement therapy. Postoperatively persistent or recurrent elevated FT4 and/or T3 with suppressed TSH levels indicated hyperthyroidism. Euthyroidism was defined as a serum TSH within the normal range.

All patients were sent a questionnaire to ask them about their current need for thyroid hormone supplementation and the date on which it had been started. If patients did not return the questionnaire, general physicians or local endocrinologists were contacted to learn more about the patient's current thyroid status and thyroid hormone use.

Each patient had a variable followup schedule, depending on the postoperative endocrine status, physician's opinion, and patient preference. The followup was defined as the interval between thyroid surgery and the date on which the last known information on the thyroid functional status was retrieved.

### 2.3. Statistical Analysis

The statistical package SPSS (version 16.0.2; SPSS, Inc., Chicago, IL) was used. Results are reported as median and range for interval variables. For nominal variables, numbers and percentages are given.

## 3. Results

### 3.1. Patient Characteristics (Table 1)

A total of 64 patients with a subtotal thyroidectomy for Graves' hyperthyroidism were identified. Two patients were excluded because of histologically demonstrated malignancy of the thyroid gland requiring additional treatment. Therefore, 62 patients were analyzed.

Of the patients studied, 56 were women (90.3%) and 6 were men (9.7%), who were 34.5 (19–63) years of age at the time of surgical intervention. The interval from diagnosis to surgical intervention was 26.5 (3–112) months. The most frequent indication for surgery was failure of medical therapy (*n* = 54, 87.1%). Patients were considered to have failed medical therapy if they were incompliant (*n* = 6, 9.7%), had an allergic reaction to medication (*n* = 7, 11.3%), or had no resolution or even recurrence of symptoms, after a course of therapy with either antithyroid medication, radioiodine or both (*n* = 41, 66.1%). All patients taking part in this study received previous medical treatment consisting of antithyroid drugs (ATD), alone or combined with levothyroxine. The antithyroid drugs were administrated for 4 to 73 months, with a median of 23 months. *β*-adrenergic blocking drugs were given in all patients for symptomatic relieve. Radioiodine therapy had been given to 13 (21.0%) of the 62 patients in the years before the operation. 17 patients (27.4%), who did not achieve euthyroidism as an outpatient with regular antithyroid medication due to noncompliance, resistance, or intolerance, were admitted and clinically treated with amiodarone, ipodate, or Lugol's solution preceding surgery. 

### 3.2. Long-Term Postsurgical Followup

Patients had a post-operative followup of 54.6 (2.1–204.2) months. Hypothyroidism occurred in 52 (83.9%) patients, 1.3 (0.2–34.2) months after surgery. In three patients postoperative hypothyroidism was diagnosed based on a high TSH with a normal free T4. From these 52 patients, the thyroid hormone status was based on recent data in 42 patients. We were unable to trace the remaining 10 patients, who were no longer with their last known general practitioner and whose current address was unavailable. These patients were discharged from further followup at the outpatient clinic 16.3 (0.7–69.7) months after surgery and 15.7 (2.1–70.9) months after being diagnosed with postoperative hypothyroidism. Four patients remained euthyroid during post-operative followup. Information on the thyroid status from the 4 euthyroid patients was retrieved 62.6 months after surgery (range 6.1–96.4 months). Six patients exhibited recurrent or persistent hyperthyroidism during the postoperative followup. The median time between the surgical treatment and the presence of hyperthyroidism was 9.7 months (range 1.1–86.3 months).

### 3.3. Surgical Complications

Permanent hypoparathyroidism was present in 2 (3.2%) patients. Only 1 (1.6%) patient developed permanent recurrent laryngeal nerve injury. There was no surgical mortality.

## 4. Discussion

This study demonstrates that the majority of patients with Graves' disease (83.9%) developed thyroid hormone deficiency after subtotal thyroidectomy. In addition, hyperthyroidism persisted or recurred in almost 10% of patients, whereas euthyroidism was established in only 6.5% of patients.

We compared our results to previously published studies evaluating thyroid status after subtotal thyroidectomy for Graves' disease (Table 2). The reported postoperative thyroid function outcomes vary, with hypothyroid rates ranging from 21.1% [6] to 83.3% [3]. The incidence of hypothyroidism in the present study seems to be relatively high compared to these studies, although two studies carried out in 2005 presented hypothyroid rates of 72.3% [7] and 83.3% [3]. Our recurrence rate of almost 10% is comparable to previously published data, with recurrence rates ranging from 0% [11, 12] to 15% [13]. The presence of recurrence in the thyroid remnant makes subsequent medical, surgical, or radioactive treatment necessary, with potential additional risks. This is reflected in the recently published guideline from The American Thyroid Association and The American Association of Clinical Endocrinologists on treatment of hyperthyroidism [2]. If surgery is chosen as primary treatment for Graves' hyperthyroidism, the guidelines recommend near-total or total thyroidectomy as procedure of choice, due to the high persistence and recurrence rates following subtotal thyroidectomy.

The highest percentage of patients with euthyroidism is reported by Chi et al. [5] and Le Clech et al. [14], 71.8% and 70.3%, respectively. One possible explanation for the variation in postoperative thyroid status rates is a varying amount of thyroid tissue left behind. The reported estimated weight of the thyroid remnant ranged from 1 gram on one side [3] to 6-7 grams in total [15]. Twenty-one percent of our patients underwent radioactive iodine therapy in the years preceding the surgery. This may have contributed to the relatively high postoperative hypothyroidism rate in our study. Unfortunately, information on radioactive iodine treatment was not reported in other studies. Another possible explanation for the variation in reported rates of postoperative hypothyroidism may be variation in the study populations, for example, in terms of sex, age, and disease severity. The fact that our hospital serves as an academic referral center presumably introduces bias towards a more severely ill and therapy-resistant patient group. Therefore, the main indication for surgery in our study was failure of medical treatment. This was also reported by another study with a relatively high rate of postoperative hypothyroidism [16]. Finally, the duration of postoperative followup varied considerably between studies, ranging from several months in some studies to several years in others. Most patients who became hypothyroid in our study tended to do so early. Because only a limited number of patients received pre-operative preparation with iodine-containing medication, this probably does not explain the high hypothyroid rates found in this study.

The main reasons to perform a subtotal thyroidectomy are to prevent postoperative hypothyroidism and complications associated with the total procedure. Given that euthyroidism was achieved in only a minority of cases, the only remaining argument in favor of STT is the assumed lower complication rate. In our series, permanent hypoparathyroidism was present in 3.2% of our patients. Permanent hypoparathyroidism has been reported to range from 0.6% [5] to 3.8% [17] in subtotal thyroidectomies. Recurrent laryngeal nerve paralysis occurred in 1.6% of our patients, comparable with reported rates ranging from 0% [5] to 1.9% [18]. In this study we did not compare STT and TT in terms of permanent complication rates. Previously published studies have shown that the risk of permanent complications with TT is no greater than that with STT [7–10].

In conclusion, in our series on subtotal thyroidectomy for Graves' hyperthyroidism euthyroidism was achieved in only a small proportion of patients. The majority of patients became hypothyroid after surgery, and approximately 10 percent had recurrent or persistent hyperthyroidism requiring additional treatment. These findings highlight the importance of continuing systematic evaluation of treatment results to optimize patient counseling. Our data favor total over subtotal thyroidectomy as the preferred surgical treatment for Graves' hyperthyroidism.

## Figures and Tables

**Table 1:** Patient characteristics.

Characteristic	*N* (%) or median (range)
Age at diagnosis (years)	32 (11–63)
Age at surgery (years)	34.5 (19–63)
Time between diagnosis and surgery (months)	26.5 (3–112)
Sex	
Female	56 (90.3%)
Male	6 (9.7%)
Surgical indication	
Failed medical treatment	54 (87.1%)
Mechanical obstruction	2 (3.2%)
Cosmetic	2 (3.2%)
Patient preference/refusal radioactive iodine treatment	2 (3.2%)
Cold nodule	1 (1.6%)
Pregnancy wish	1 (1.6%)
Preoperative radioactive iodide treatment	
No	49 (79.0%)
Yes	13 (21.0%)
Previous medical treatment	
Antithyroid drugs (+ thyroxine + *β*-blocker)	62 (100%)
Duration of thyrostatic treatment(months)	23 (4–73)
Medical treatment at the time of surgery	
Antithyroid drugs	45 (72.6%)
Antithyroid drugs + amiodarone, Lugols or ipodate	11 (17.7%)
Amiodarone	1 (1.6%)
Ipodate	5 (8.1%)

**Table 2:** Thyroid function after subtotal thyroidectomy for Graves' disease.

Study	Type of surgery	No. of patients	Followup	Postoperative thyroid function (%)		
				Hypothyroid	Euthyroid	Hyperthyroid
Andaker et al. [12] (1992)	BST	23	3.6 (3-4) years	34.8	60.9	4.3
	STT	27	3.6 (3-4) years	44.4	55.6	0
Chi et al. [5] (2005)	BST	166	26.4 ± 1.1 months	21.1	69.9	9.0
	STT	174	26.4 ± 1.1 months	26.4	71.8	1.7
Chou et al. [19] (1999)	BST	205	26.9 ± 15 (9–97) months	44.2	48.7	7.0
Ku et al. [7] (2005)	BST	119	65 (6–104) months	72.3	21.8	5.9
Kuma et al. [15] (1991)	STT	67	8.8 ± 1.5 years	32.9	56.7	10.4
Lal et al. [3] (2005)	STT	30	6–120 months	83.3	10.0	6.7
Le Clech et al. [14] (2005)	STT	312	?	23.7	70.3	6.0
Lepner et al. [11] (2008)	STT	21	49 (24–70) months	66.7	33.3	0
Miccoli et al. [17] (1996)	STT	80	6–48 months	46.3	46.3	7.5
Moreno et al. [16] (2006)	BST	202	5 years	62.1	35.5	2.4
Robert et al. [20] (2001)	STT	56	7 (1–15) years	73	23	4
Sivanandan et al. [13] (2004)	BST	213	5 years	28.2	56.8	15.0
Sugino et al. [21] (1995)	STT	728	42.5 (24–58) months	46.4	39.0	14.6
Present study (2010)	STT	62	55 (2–204) months	83.9	6.5	9.7
